# A delayed presentation of an intracranial haemorrhage as the result of a full-thickness electrical brain injury: Illustrative case

**DOI:** 10.1016/j.cjtee.2025.03.004

**Published:** 2025-12-31

**Authors:** Jesse George Kwete Bulabula, Moses Asante-Bremang, Anthony A. Figaji, Nqobile S. Thango, Patricia SN. Kambinga, Johannes MN. Enslin

**Affiliations:** a Division of Neurosurgery, University of Cape Town, Cape Town, Republic of South Africa; b Department of Neurosurgery, Red Cross War Memorial Children's Hospital, Cape Town, Republic of South Africa

**Keywords:** Electrical brain injury, Electrical burn, Electrocution injury, Traumatic brain injury, Delayed intracerebral haemorrhage

## Abstract

Electrical brain injuries are rare and often under-reported, with most documented cases involving secondary mechanical trauma. Isolated electrical injuries to the brain remain poorly characterised. We report a case of 2-year-old girl who sustained an isolated full-thickness electrical brain injury without associated mechanical trauma. Clinical findings, neuroimaging, and surgical management were reviewed to highlight the progression and complications observed. This report aims to describe the delayed sequelae, pathophysiology, and management challenges of a full-thickness electrical brain injury in a child, and illustrates the complex and delayed pathophysiology of electrical brain injuries, in which venous thromboembolic mechanisms may contribute to secondary deterioration. Aggressive debridement of ischemic tissue during surgical intervention may improve wound healing and overall outcomes.

## 1 Introduction

Despite traumatic brain injuries being a leading cause of disability and death, very little literature exists describing traumatic brain injuries due to electrical burn injuries.^1^ Electrical burn injuries are uncommon, comprising 0.04%–5% of burn unit admissions in developed countries, and up to 27% in developing countries.^2^ Access to electricity in South Africa remains a challenge, with households relying on unsafe, informal connections. These pose serious risks, including electrical burns and fires. Our patient sustained an isolated electrical brain injury due to such an incident.^3^

The clinical effects of electric injuries are well described, but with limited neurological understanding. Immediate manifestations of electrical injuries can be life-threatening, including cardiac and respiratory arrest, rhabdomyolysis, acute kidney injury, loss of consciousness, seizures, motor and sensory deficits, and altered Glasgow coma scale (GCS). People survive the acute manifestations and then endure late complications days to months following the initial insult. Delayed focal deficits include cerebral (hemiplegia, aphasia), spinal (transverse myelitis, progressive muscular atrophy, amyotrophic lateral sclerosis), and peripheral nerve (neuropathies, radiculopathies). Delayed non-focal symptoms present as cerebellar ataxia, psychoneurotic behaviour, personality changes, confusion, amnesia, and headaches, and their occurrence is not limited to cases in which the brain lies in the electric current pathway.^4^, ^5^, ^6^

This case report describes a 2-year-old girl who sustained a delayed, extensive intracranial haemorrhage consequential to high voltage electrical contact with her head. We also describe the nuances and challenges faced whilst managing the patient, focusing on the pathogenies of delayed electrical intracranial haemorrhage. To our knowledge, no other case has been described in the literature.

## 2 Case report

### 2.1 History and presentation

**Fig. 1:**
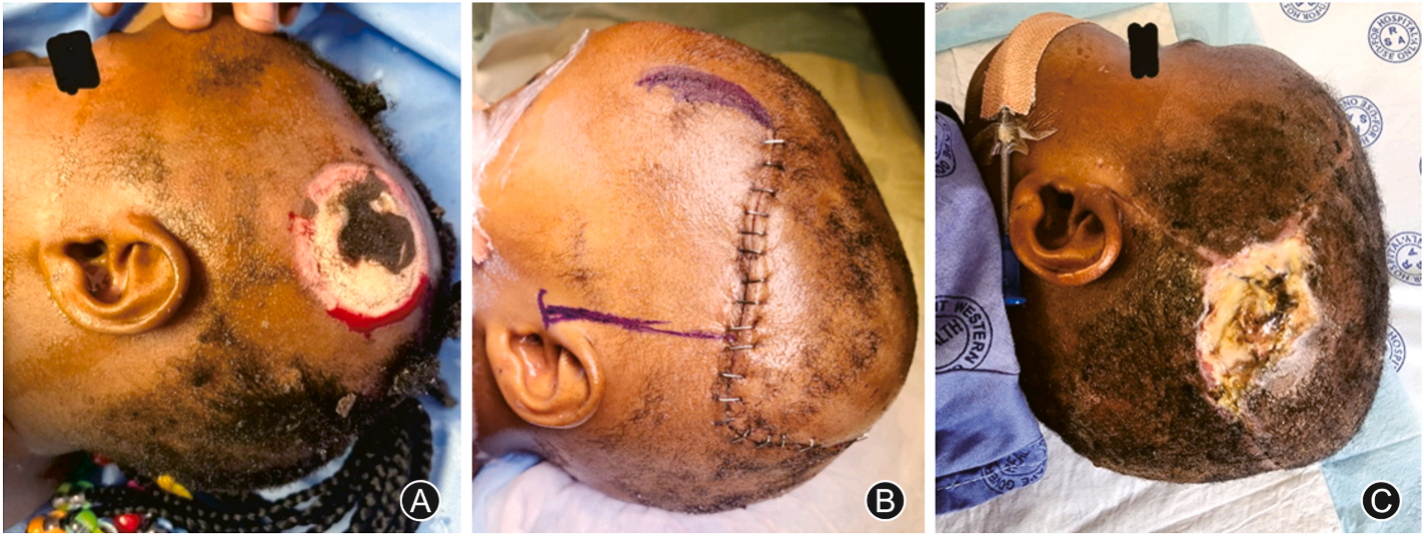
(A) Left parietal 5 × 5 cm full-thickness electrical burn. (B) Rotational scalp flap staples with marked craniotomy incision. (C) Surgical wound dehiscence site, day 21 post craniotomy.

**Fig. 2:**
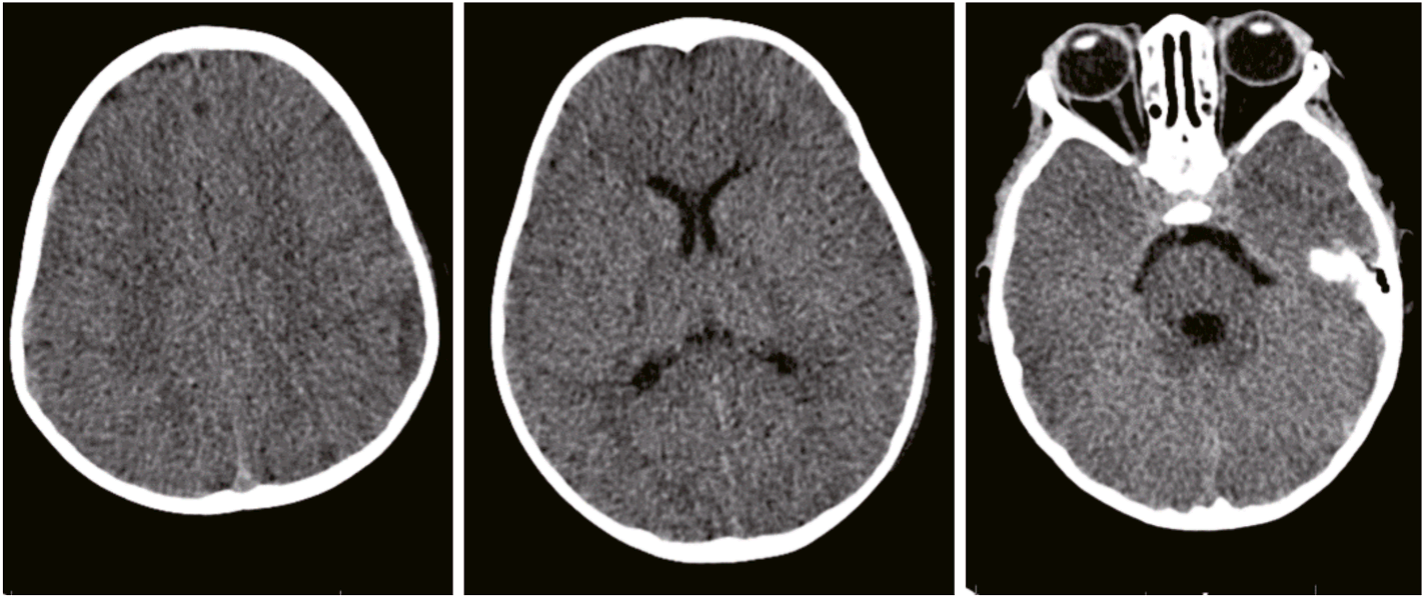
Initial axial uncontrasted CT of the brain showing left parietal hypodensity underlying the clinical area of the full-thickness scalp burn wound.

A 2-year-old girl, otherwise well with no prior medical history, sustained an electrical burn injury whilst playing at home. The child's calvarium had accidently contacted a corrugated iron wall attached to an informal electricity connection. The patient was found unresponsive and presented to the paediatric emergency unit at Red Cross War Memorial Children's Hospital in status epilepticus. Clinically, the patient had a blood pressure of 96/60 mmHg, a pulse of 160 beats/min, a respiratory rate of 23 breaths/min, and a temperature of 37.9 °C. The patient sustained an electrical burn wound of 5 × 5 cm, full thickness, to her left parietal scalp (Fig. 1A). Two other deep partial thickness burn wounds were noted, including a 3 × 2 cm left wrist and a 3 × 3 cm right lateral malleolar wound. The total body surface area of electrical burns was 5%. On presentation, the patient had a GCS of 3 (E1M1V1), reactive pupils at 3 mm with no other clinical-, neurological-, or biochemical-abnormalities. No other injuries were noted on the trauma primary and secondary survey. Electrocardiography was in sinus rhythm with no conduction abnormalities. An uncontrasted CT brain scan was done, which showed a left parietal hypodensity underlying the clinical area of the full-thickness scalp burn wound (Fig. 2).

### 2.2 Course/management

**Fig. 3:**
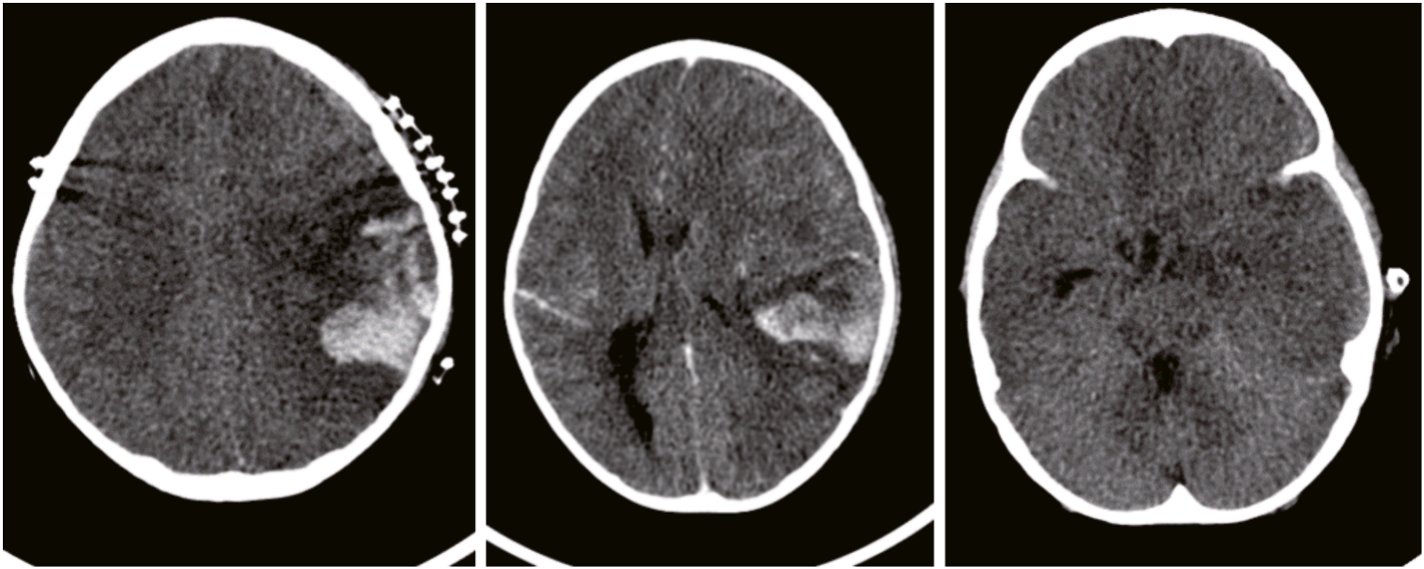
Axial contrasted CT of the brain showing diffuse brain injury with left temporal-parietal intracranial haemorrhage and associated mass effect.

**Fig. 4:**
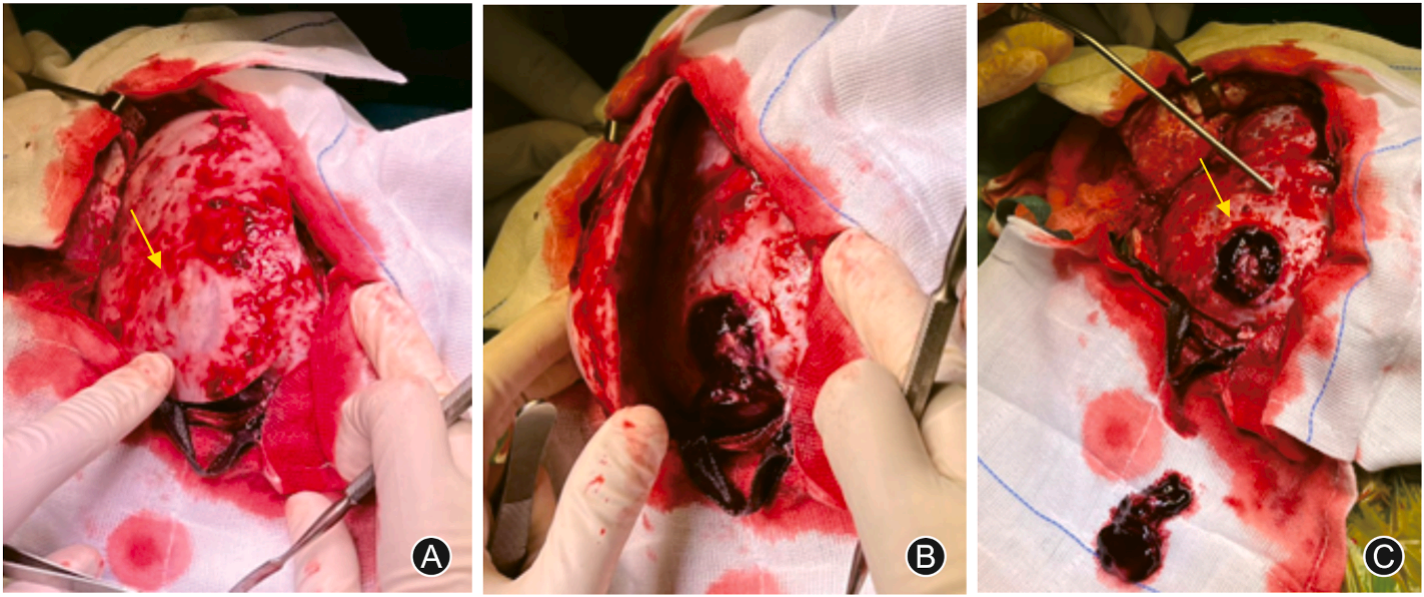
(A) Intraoperative finding of a denatured area of the cranium indicated by the yellow arrow. (B) Intraoperative findings of denatured bone underlying the dural defect. (C) A 5 × 5 cm dural defect is indicated by the yellow arrow.

**Fig. 5:**
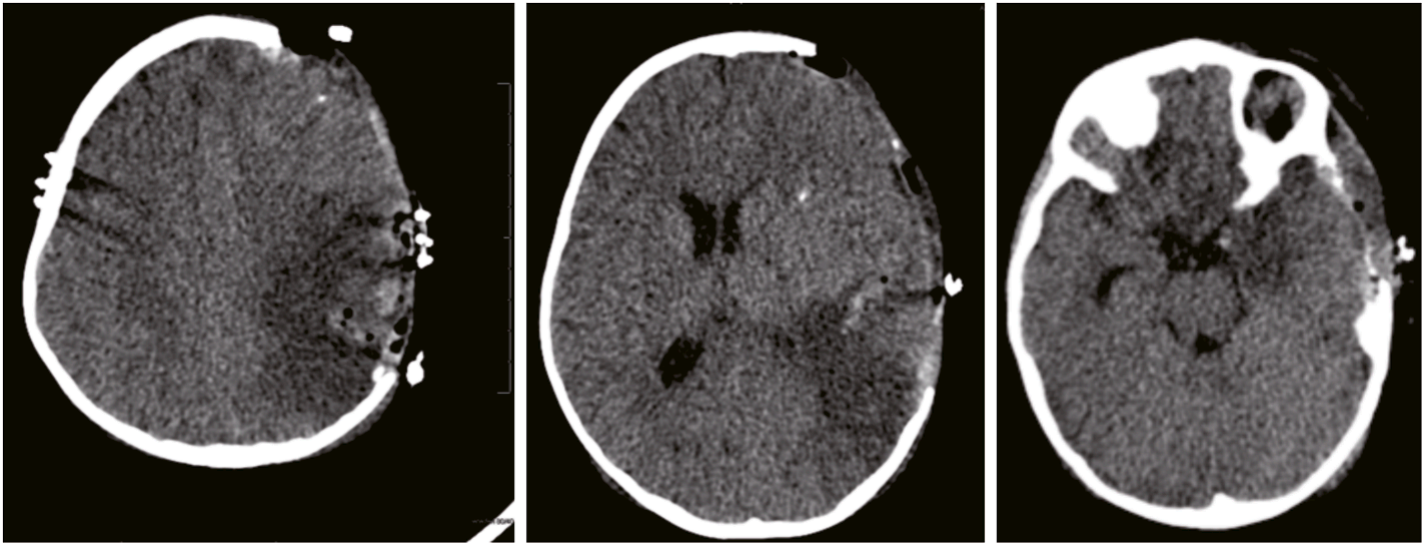
Axial post-operative decompressive craniectomy, evacuation of ICH and insertion of ICP monitors. ICH: intracranial haemorrhage, ICP: intracranial pressure.

**Fig. 6:**
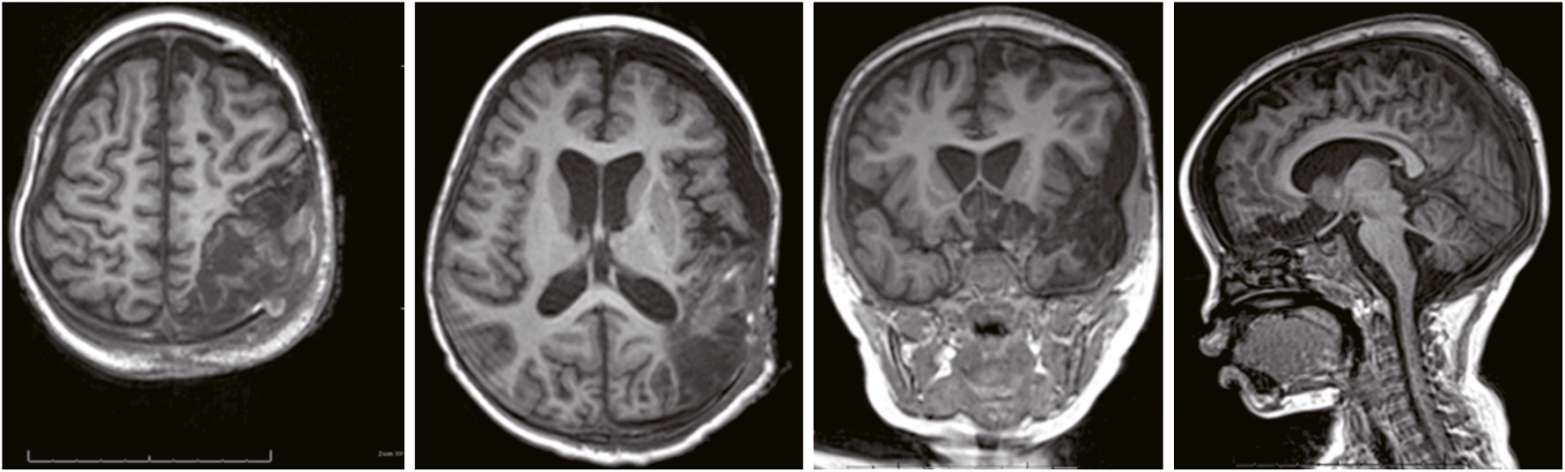
MRI showing a large multi-territorial infarct involving the majority of the left cerebral hemisphere and medial aspect of the right frontal lobe with established encephalomalacia and atrophy. There are blood products of different ages in the left parietal lobe.

The patient was intubated on arrival to the emergency unit, and status epilepticus was aborted with Levetiracetam and Phenobarbitone in the paediatric intensive care unit (ICU). After 2 days of ventilation in the ICU, the patient was extubated and discharged to the general paediatric ward with a GCS of 15/15 and no neurological deficits. She was walking, eating, and talking as normal. On day 7 post-injury, the paediatric burn unit performed a debridement of the burn wounds with a rotational scalp flap (Fig. 1B), with autograft harvested from the patient's right thigh. It was noted intra-operatively that the full-thickness burn involved the underlying calvarium, but with no visible bony defects. The patient was asymptomatic in the ward, and the wound was covered with a low suction vacuum dressing. Day 9 post-injury, with no new history of trauma, the patient experienced generalised tonic-clonic seizures. This was after 7 days of being seizure-free on anti-epileptics. Her level of consciousness deteriorated to a GCS of 7 (E2M4V1) and was re-intubated and ventilated in the ICU. A contrasted CT brain scan was done and showed a significant parietal intracranial haemorrhage with a subdural hematoma and a 6 mm midline shift, where the prior parietal hypodensity was visualised on the initial scan (Fig. 3). The Patient was taken for an urgent decompressive craniotomy, evacuation of haematomas, dural expansion graft, and intracranial pressure (ICP) monitor insertion. Intra-operatively, it was noted that there was a 5 × 5 cm area of denatured cranium, which correlated with an underlying “punched out” dural defect of the same size (Fig. 4). Only minimal bleeding was noted within the brain; however, large zones of necrotic tissue were identified and surgically debrided. A pericranium dural graft was used, and a watertight duraplasty was performed. No dura was resected at this time, only grafted. A postoperative scan showed adequate evacuation with reduction in midline shift and adequate bony decompression (Fig. 5). The patient underwent ICP monitoring and regulation, with ICP being well controlled throughout ICU admission, being maintained under 20 mmHg with central perfusion pressures above 50 mmHg. Patient was extubated after 9 days of ICP regulation and discharged to the neurosurgery ward for further rehabilitation and wound care. An inpatient MRI brain scan was done on day 28 after the initial injury and showed evolution of the prior noted infarcts. The extent of the diffuse brain injury was also visualised, revealing involvement of the right midbrain, pons, left cerebral peduncle and left basal ganglia. A left subdural collection was noted with blood products of different stages as depicted below (Fig. 6). The cerebral oedema had resolved with no midline shift present (Fig. 6). After a week of rehabilitation in the ward, the patient developed wound dehiscence at the site of the left craniectomy on day 21 post-craniectomy (Fig. 1C). The patient was then taken for wound debridement and a rotational flap. Intra-operative, a dural breach was noted at the prior duraplasty site where the initial full-thickness burn had occurred. The dural edges were then resected widely, and a new autologous fascia lata graft was applied. This duraplasty-necrosis area resembled the previous “punched out” dural lesion closely and seems to have been exactly at the edges of the previous dural defect that was left intact at the time of the duraplasty. There was no cerebrospinal fluid leak noted, but due to a dural breach with open communication from the area of wound dehiscence, the patient completed 10 days of meropenem.

The left craniectomy wound granulated well, and the child was discharged home (2 months after initial injury) with the aid of a multidisciplinary team, with a GCS of 15 and no subsequent seizures. Neurologically patient had a 4 out of 5 power hemiparesis in her right upper and lower limbs, and power on the left-hand side was intact. The patient was able to stand and walk with assistance. The speech had improved with the patient being able to say a few phrases, and the child was tolerating oral feeds. The patient was reviewed at the neurosurgery clinic 1 month following discharge with a healed craniotomy scar, subtle right hemiparesis, appropriate developmental milestones, and seizure-free. There is still a bony defect, as we have not replaced the bone yet, due to problems with wound dehiscence. The patient is planned to undergo cranioplasty 6 months following initial craniectomy and to continue outpatient rehabilitation.

## 3 Discussion

### 3.1 Proposed pathogenesis

The intracranial effects of high voltage injuries are poorly understood. Our patient initially presented with a parietal infarct underlying the full-thickness burn, which then progressed to an intracranial haemorrhage 9 days later. In the setting of electrical injuries, the following theories of intracranial pathophysiology exist: the direct thermal effect of the current, direct electrolytic effect of the electrical charge, actual mechanical trauma at the time of electrical injury, and intense peripheral vasoconstriction causing infarcts and thrombo-embolic events.^4^^,^^7^ The complications of electrical intracranial trauma can be divided into immediate and delayed. Basal ganglia haemorrhages, thalamic haemorrhages, cerebral oedema, and even hydrocephalus have been reported as immediate manifestations following high voltage electrical injuries.^4^^,^^5^^,^^7^ Non-electrical delayed traumatic intracranial haemorrhages have been documented, though the precise underlying mechanisms remain poorly understood. Proposed pathophysiological processes include local or systemic coagulation abnormalities secondary to major sinus occlusion, vascular necrosis within injured brain regions, transient cerebral dysautoregulation, and intracranial pressure reduction through either medical or surgical intervention.^8^ Determining the extent of pathological overlap between electrical and non-electrical delayed traumatic intracranial haemorrhages remains challenging. However, this case serves as a pivotal reference for exploring this concept.

A delayed intracranial haemorrhage secondary to an electrical injury has never been documented. In this case, the initial infarct is likely attributable to the aforementioned mechanisms, with vasoconstriction playing a predominant vascular role. Subsequent haemorrhage and delayed manifestations may be attributed to the progression of a vascular dissection causing an intracerebral haemorrhage. Some case reports have shown that delayed sequelae may present due to cortical venous thrombosis or venous sinus thrombosis, with a plausible theory being that current passing through the vessels disrupts Virchow's Triad by means of endothelial vessel damage and vasoconstriction, which results in clot propagation and thrombo-embolic events.^9^^,^^10^ Progressive infarcts leading to haemorrhagic conversion are another plausible theory. Haemorrhagic strokes evolve from ischemic strokes due to blood products extravasating across a disrupted blood-brain barrier.^11^ Intra-operatively, a dural defect was noted in keeping with the dimensions of the overlying denatured bone and full-thickness scalp burn. This potentially indicates an area of cerebral parenchyma being exposed to electrical current, devoid of normal protective cerebral barriers, subsequently denaturing and haemorrhaging. These events may result in cerebral parenchymal and vascular damage, leading to widespread blood-brain barrier disruption and diffuse brain injury.

We believe the delayed haemorrhage in this patient is most likely driven by venous thrombosis and its subsequent complications. Small veins and venules do not have encompassing smooth muscle cells, making veins more susceptible to thrombosis.^12^ Studies in mice revealed that microthrombi occluded up to 70% of all investigated venules and 33% of arterioles after traumatic brain injuries.^11^ Studies in rats showed that despite endothelial rolling of leukocytes in arterioles and venules, leukocyte-platelet aggregates were only observed in post-capillary venules, providing more evidence for the susceptibility of veins to thrombosis.^12^ The venous component of the blood-brain barrier has proven more susceptible to breakdown in other intracranial pathologies due to an increase in pial venular pressure, diameter and resultant endothelial dysfunction.^12^ The presence of increased arterial inflow with decreased venous outflow can cause venous congestion, which in turn can lead to rupture of friable, thin-walled cortical veins, causing intracranial haemorrhages. The final indication pointing to a venous thromboembolic cause is the delayed onset of the haemorrhage. Traumatic arterial haemorrhages are generally acute, whilst delayed bleeds, as in our case, are often seen in venous intracranial bleeds.^8^

### 3.2 Nuances in management

The delayed sequelae of the electrical injury posed multiple challenges in the management of this patient. The initial CT scan showed no significant injury apart from a small parietal infarct, while a subsequent scan following a second seizure revealed an intracranial hematoma in the same region as the original infarct, suggesting progression of the electrical injury or, less likely, an additional head injury from the second seizure. In hindsight, early cerebral vascular imaging by means of a CT angiogram, digital subtraction angiography, or magnetic resonance angiography (MRA) could have been instrumental in identifying the mechanisms behind the delayed haemorrhage. Early identification might have prevented the haemorrhage and avoided the surgery. Unfortunately, vascular imaging was deferred due to the acute deterioration before surgery. Later, MRA imaging revealed the full extent of the injury, which was not visible on the initial CT scan, though no vascular pathology was noted on this delayed MRI.

An additional delayed complication of the electrical injury was wound breakdown, coupled with the failure of the duraplasty. In electrical injuries, the extent of microscopic damage often extends beyond the immediate contact zone.^13^ In retrospect, during the initial decompressive craniectomy and duraplasty, a more extensive circumferential dural debridement might have been beneficial to address the denatured dura, which would not have been apparent macroscopically. A more aggressive durotomy could have potentially facilitated improved wound healing and prevented subsequent dehiscence.

### 3.3 Knowledge gaps

Our patient had a relatively good outcome given the nature of the delayed presentation and significant intracranial haemorrhage resulting in increased ICP. However, there is still great difficulty in determining the long-term effects of severe electrical injuries on her developing brain. Despite all the above-mentioned clinical neurological presentations, there are no large cohorts of patients with isolated electrical brain injuries to make definitive conclusions regarding management and prognosis. All existing evidence is pooled from case reports or small retrospective cohorts of patients with electrical injuries in which confounding factors are not accounted for. In conclusion, this case highlights the complexities of delayed intracranial haemorrhage following high-voltage electrical injury, a phenomenon not previously documented in the literature. The proposed mechanisms, including venous thrombosis, progressive infarction, and blood-brain barrier disruption, emphasise the unique pathophysiology of electrical brain injuries. The challenges encountered in diagnosis and management, particularly the limitations of early imaging and the need for more aggressive dural debridement, underscore the necessity of further research. Given the lack of large patient cohorts, this case serves as a valuable reference for understanding the neurological, radiological, and surgical nuances of electrical brain injuries, contributing to improved clinical decision-making and patient outcomes.

## 4 Conclusion

Despite the difficulties in electrical brain injuries, the following recommendations can be derived from our case report: (1) Early MRI brain allows better visualization of the extent of the injury, which may aid in determining long-term prognosis and rehabilitation requirements. (2) Cerebrovascular angiography in the presence of hypodensities or neurological deficits by means of a CT angiography or MRA may assist in detecting thrombo-embolic events and, if detected early, can prevent progression of cerebral ischemia, and provide further knowledge on the pathophysiology of electrical brain injuries. (3) Wider margins of necrotic tissue debridement at the layer of scalp, calvarium, and dura could potentially facilitate wound healing, as the extent of denatured tissue from the initial injury may not be macroscopically visible.

This case report is unique in that it is the first case of a delayed presentation of an isolated electrical burn injury, providing further evidence of the pathophysiology of thrombo-embolic phenomena being the driving factor of ischemia following electrical brain injuries. However, further cases and cohorts with cerebrovascular imaging are needed to affirm this theory.

## CRediT authorship contribution statement

**Jesse George Kwete Bulabula:** Conceptualization, Software, Resources, Project administration, Methodology, Investigation, Formal analysis, Data curation, Writing – review & editing, Writing – original draft, Visualization. **Moses Asante-Bremang:** Methodology, Data curation. **Anthony A. Figaji:** Writing – review & editing, Supervision. **Nqobile S. Thango:** Writing – review & editing, Supervision. **Patricia SN. Kambinga:** Methodology, Data curation. **Johannes MN. Enslin:** Writing – review & editing, Writing – original draft, Supervision, Conceptualization.

## Ethical statement

Informed consent has been obtained, and all data were confidential except for academic communication.

## Funding

This research did not receive any specific grant from funding agencies in the public, commercial, or not-for-profit sectors.

## Declaration of competing interest

During the preparation of this work the author(s) used Chat GPT to edit language. After using this tool/service, the author(s) reviewed and edited the content as needed and take(s) full responsibility for the content of the publication.
